# Transcriptomic analysis of PADI4 target genes during multi-lineage differentiation of embryonic stem cells

**DOI:** 10.1098/rstb.2022.0236

**Published:** 2023-11-20

**Authors:** Anup Kumar Singh, Soumen Khan, Daniel Moore, Simon Andrews, Maria A. Christophorou

**Affiliations:** ^1^ Epigenetics, Babraham Institute, Cambridge CB22 3AT, UK; ^2^ Bioinformatics Facility, Babraham Institute, Cambridge CB22 3AT, UK

**Keywords:** embryonic stem cells, lineage specification, peptidylarginine deiminase

## Abstract

During mammalian embryo development, pluripotent epiblast cells diversify into the three primary germ layers, which will later give rise to all fetal and adult tissues. These processes involve profound transcriptional and epigenetic changes that require precise coordination. Peptidylarginine deiminase IV (PADI4) is a transcriptional regulator that is strongly associated with inflammation and carcinogenesis but whose physiological roles are less well understood. We previously found that *Padi4* expression is associated with pluripotency. Here, we examined the role of PADI4 in maintaining the multi-lineage differentiation potential of mouse embryonic stem (ES) cells. Using bulk and single-cell transcriptomic analyses of embryoid bodies (EBs) derived from *Padi4* knock-out (*Padi4-KO*) mouse ES cells, we find that PADI4 loss impairs mesoderm diversification and differentiation of cardimyocytes and endothelial cells. Additionally, *Padi4* deletion leads to concerted downregulation of genes associated with polarized growth, sterol metabolism and the extracellular matrix (ECM). This study indicates a requirement for *Padi4* in the specification of the mesodermal lineage and reports the *Padi4* associated transcriptome, providing a platform for understanding the physiological functions of *Padi4* in development and homeostasis.

This article is part of the Theo Murphy meeting issue ‘The virtues and vices of protein citrullination’.

## 1. Introduction

Pluripotency is the potential of stem cells to self-renew indefinitely and give rise to any differentiated cell type. During gastrulation, pluripotent epiblast cells differentiate into the three primary germ layers—endoderm, mesoderm and ectoderm—which subsequently give rise to all of the tissues and organs in the adult organism [1]. This essential developmental stage happens from embryonic day (E) 6.5 to 8.5 and is accompanied by profound transcriptional and epigenetic changes as cells adopt different identities and fates [2–5].

The peptidylarginine deiminase (PADI or PAD) enzymes catalyse the post-translational conversion of arginine protein residues to non-coded citrullines. Peptidylarginine deiminase IV (PADI4) is a predominantly nuclear enzyme with well-established roles as a transcriptional regulator, although emerging evidence implicates it also in the regulation of inflammatory signalling and the extracellular matrix (ECM) (reviewed in [6]). PADI4 becomes activated under conditions of infection and sterile inflammation, where it modulates the expression of inflammatory cytokine genes [7,8] and chromatin de-compaction [9,10]. Aberrant PADI4 expression and activity are features of various inflammatory diseases and cancers and PADI4 has been shown to act as a cofactor to key oncogenic transcriptional regulators [11–13]. Thus, PADI4 deregulation is strongly associated with the pathophysiology of inflammatory disorders, autoimmunity and cancer.

The physiological functions of PADI4 are less well understood, as are its modes of action under physiological conditions (whether through transcriptional, signalling or cell–ECM regulation). In healthy mammals, *Padi4* is most highly expressed in the blood and the bone marrow, although it does not have a cell-intrinsic role in mouse haematopoiesis [14,15]. We previously demonstrated that PADI4 is also expressed in embryonic stem (ES) cells and mediates the establishment of pluripotency during the reprogramming of somatic cells to induced pluripotent stem (iPS) cells [10]. *Padi4* is part of the pluripotency network, as its expression is regulated by the cardinal pluripotency factor Pou5f1, while *Padi4* knock-down or pharmacological inhibition lead to the downregulation of pluripotency markers such as *Nanog* and *Tcl1* [10].

Here, we examined the expression of *Padi* genes during mouse gastrulation using previously published single-cell transcriptomic data [5] and found that *Padi4* is expressed in epiblast cells from E6.5. To understand whether PADI4 has a role in maintaining the full differentiation potential of stem cells, we interrogated the transcriptional and cellular consequences of *Padi4* loss in multi-lineage differentiation, using an embryoid body (EB) differentiation system. This system involves the culture of ES cells in three-dimensional spheroid aggregates, which results in their differentiation into cells of the three primary germ layers and faithfully recapitulates the cellular and transcriptional changes that occur during early embryo development *in vivo* [16,17]. Using bulk RNA-sequencing analyses, we show that *Padi4* knock-out (*Padi4-KO*) ES cells are impaired in the upregulation of genes associated with polarized growth and vessel formation, genes involved in sterol metabolism and genes associated with the ECM. Using single-cell RNA sequencing analyses we show that *Padi4-KO* EBs are impaired in the differentiation of cells of the mesodermal lineage, cardiomyocytes and endothelial cells. These results indicate that PADI4 has a role in mesoderm specification during gastrulation. The PADI4-dependent transcriptome reported here may provide a basis for further studies into the roles of PADI4 in cell physiology, mammalian development and homeostasis.

## 2. Results

### (a) Analysis of *Padi* gene expression during mouse gastrulation

To understand the expression pattern of *Padi4* during mouse embryo development we interrogated previously published single-cell RNA sequencing data of mouse gastrulation [5]. *Padi4* is most highly expressed in the epiblast, which comprises the pluripotent cell compartment, and its expression is detected from E6.5 (electronic supplementary material, figure S1). As embryos progress through gastrulation (E7–E8), sporadic *Padi4* expression is detected within the primitive streak, the extra-embryonic lineages (extra-embryonic ectoderm and endoderm), the caudal epiblast and caudal mesoderm, while strong expression is detected in some gut cells and differentiated cardiomyocytes by E8.5 (electronic supplementary material, figure S1).

The expression of the other *Padi* family genes (*Padi1, Padi2, Padi3* and *Padi6*), all of which show different expression patterns during this developmental window, is presented in electronic supplementary material, figures S2–S5. *Padi1* and *Padi3* are widely expressed across the different stages of gastrulation (electronic supplementary material, figures S2 and S4). Both PADI1 and PADI3 are expressed in the adult skin and have been implicated in skin homeostasis [18], so we were surprised to find strong *Padi3* expression across the mesenchyme and blood lineages. These data suggest that *Padi3* has a yet unexplored role in blood development. PADI1 was previously been implicated in pre-implantation development, with PADI1 inhibition leading to arrest at the four-cell stage [19]. The observed expression of *Padi1* across the epilast and primitive streak suggests that it may also regulate aspects of embryonic development post implantation. *Padi2*, the ancestral member of the *Padi* gene family, is most widely expressed in adult tissues and, along with *Padi3,* was shown to maintain the stem cell state of trophoblast stem cells [20]. However, *Padi2* is very sparsely expressed during gastrulation (electronic supplementary material, figure S3), suggesting it is unlikely to operate at the early lineage commitment stage of development. *Padi6,* which has well-established roles in the oocyte and is expressed highly in the pre-implantation embryo [21,22], shows very sporadic expression during gastrulation (electronic supplementary material, figure S5), suggesting that it does not have a role during this stage of development.

### (b) *Padi4* loss results in widespread transcriptional changes during embryonic stem cell differentiation

**Figure 1.**
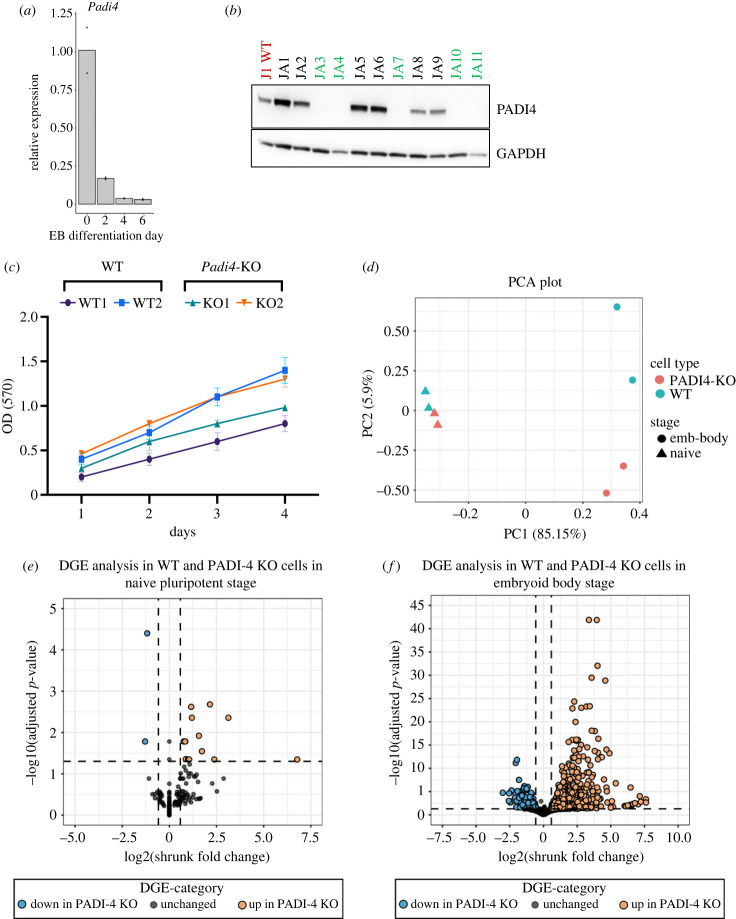

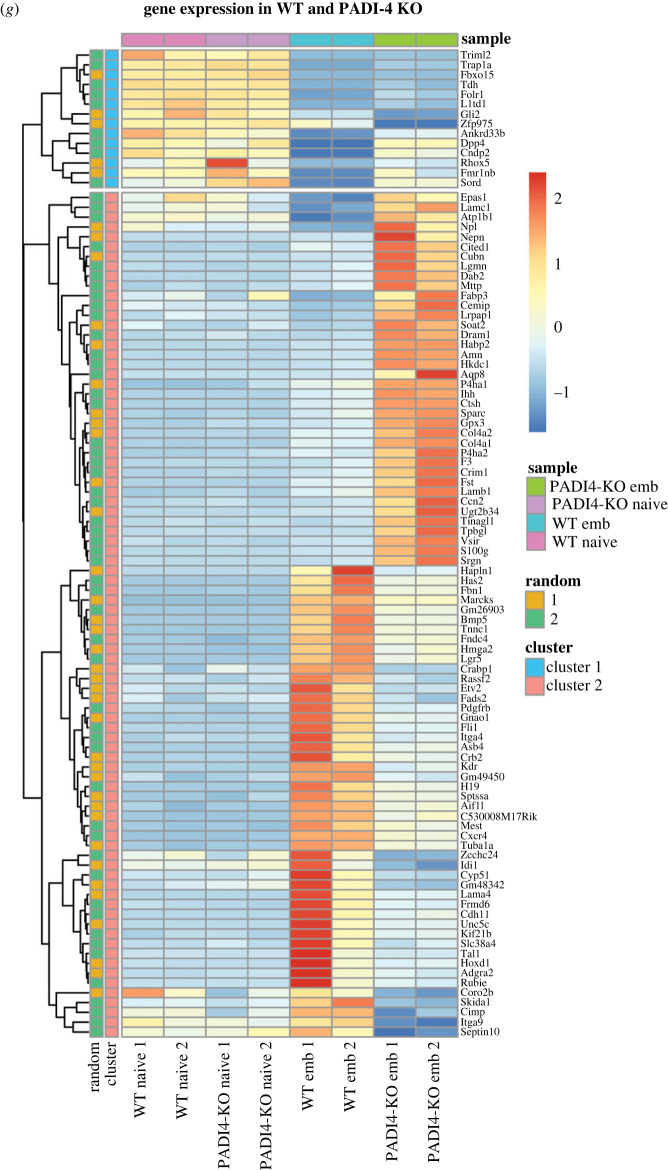
Gene expression changes resulting from *Padi4* deletion. (*a*) RT-qPCR analysis of *Padi4* expression in clonal mouse ES cell lines at the naive state (day 0) and after 2, 4 and 6 days of EB differentiation. Expression is relative to the geometric mean of *Ubqc*, *Gapdh* and *Atp5b*. Statistical analysis was not performed as inappropriate for two biological replicates. (*b*) Immunoblot analysis showing PADI4 expression in naive mouse ES cell clonal lines targeted for PADI4 deletion using CRISPR-Cas9. GAPDH presented as loading control. (*c*) MTT assay for the quantification of cell proliferation of the two WT and two *Padi4-KO* clonal lines used in RNA-sequencing analyses. (*d*) Principle component analysis (PCA) of the transcriptomic changes observed in two clonal *Padi4-KO* (red) and two clonal wild-type control (blue) cell lines, at the naive pluripotent state (triangles) or after 6 days of EB differentiation (circles). (*e*) Differential gene expression analysis of WT and *Padi4-KO* ES cells at the naive state. (*f*) Differential gene expression analysis of WT and *Padi4-KO* embryoid bodies after 6 days of differentiation. (*g*) Heat map of the top 60 differentially expressed genes. The complete dataset is presented in electronic supplementary material, file S1.

We previously demonstrated that *Padi4* expression is associated with the pluripontent cell state [10]. This agrees with the *Padi4* expression observed during mouse gastrulation (electronic supplementary material, figure S1, E6–E7.25). To assess the dynamics of *Padi4* expression during exit from pluripotency we profiled *Padi4* mRNA levels in J1 mouse ES cells during differentiation to EBs. We observe that *Padi4* levels decline quickly, with an 80% decline by day 2 and complete downregulation by day 6 of EB differentiation (figure 1*a*). These findings suggest that PADI4 has a role in pluripotent cell function. To test this, we examined the transcriptional consequences of *Padi4* loss in pluripotent stem cells and during multi-lineage differentiation. We used CRISPR-Cas9 to target *Padi4* exon 1, downstream of the transcriptional start side, in J1 mouse ES cells. A number of clonal knock-out (KO) lines were generated (figure 1*b*) while clonal wild-type (WT) control clonal lines were generated through the same process in the absence of targeting guide RNAs (gRNAs). Two WT (WT1 and WT2) and two KO lines (JA4 and JA10, named KO1 and KO2, respectively, hereafter) were chosen for further analysis. ES cell line KO1 contained indels that resulted in frame shift mutations of +8 and +1 bases, while KO2 contained frame shifts of +1 and −2 bases (electronic supplementary material, figure S6). These mutations resulted in loss of PADI4 protein expression (figure 1*b*). The *Padi4-KO* lines did not show a defect in cell proliferation (figure 1*c*).

We conducted bulk RNA-sequencing analyses of the above lines cultured in naive pluripotency conditions (2i/LIF) and after 6 days of EB differentiation. Principal component analysis (PCA, figure 1*d*, triangle points) and differential gene expression analysis (DGE, figure 1*e*) show that WT and *Padi4-KO* naive ES cells are highly similar at the transcriptional level. We observed minimal transcriptional changes, with 15 genes upregulated and two genes downregulated in this state. Conversely, bulk RNA-sequencing analysis of EBs after 6 days of differentiation shows widespread transcriptional changes in *Padi4-KO* compared to WT EBs, as demonstrated by PCA (figure 1*d*, circle points) and DGE analyses (figure 1*f*). While we observe some inherent variability between the clonal WT lines, we focused our analysis on genes that show a clear change in expression between both WT and the *Padi4-KO* lines. We observe 562 upregulated and 185 downregulated genes. An unsupervised cluster analysis of the top 60 differentially expressed genes across all conditions is shown in figure 1*g* and the full dataset is presented in electronic supplementary material, file S1. The most highly downregulated gene is *Tal1*, a key regulator of mesoderm diversification [5].

### (c) The PADI4-regulated transcriptome is enriched in genes involved in blood vessel growth, cell metabolism and the extracellular matrix

To understand the types of biological processes altered by *Padi4* loss during embryonic stem cell differentiation, we interrogated the transcriptomic data obtained from WT and *Padi4-KO* EBs using Gene Ontonogy (GO) analyses. The gene lists returned for the GO analyses described below are presented in electronic supplementary material, file S2.

**Figure 2.**
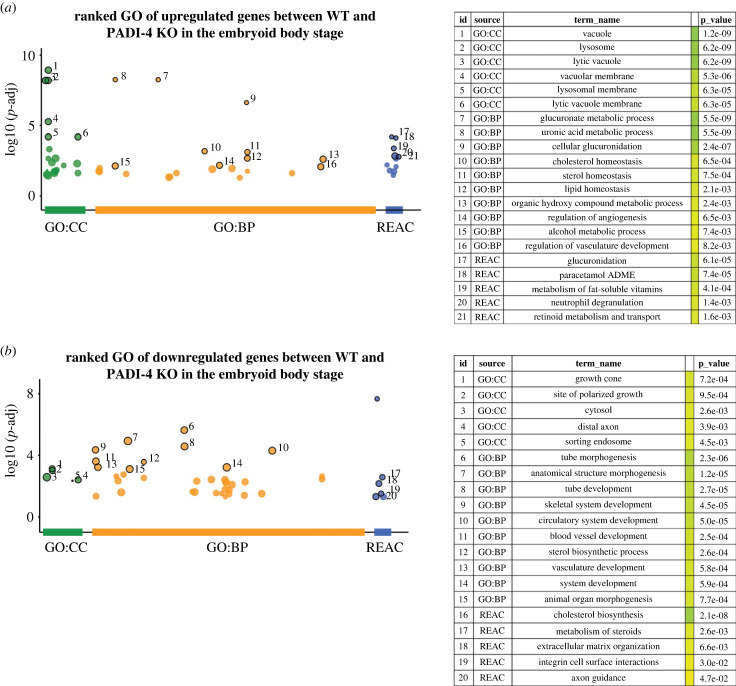
Gene Ontology analysis of the embryoid body PADI4-regulated transcriptome. (*a*) Gene Ontology for Cellular Component (GO:CC), Biological Process (GO:BP) and REACTOME pathway analysis for the genes upregulated in *Padi4-KO* EBs. (*b*) GO:CC, GO:BP and REACTOME pathway analysis for the genes downregulated in *Padi4-KO* EBs. The lists of genes identified for each category are presented in electronic supplementary material, file S2.

We considered the upregulated and downregulated genes separately, to understand which processes are impaired or aberrantly enhanced, as a result of *Padi4* loss. Considering the processes enriched within the set of upregulated genes (figure 2*a*), Gene Ontology for Cellular Component (GO:CC) shows a very strong enrichment for factors associated with lytic organelles (vacuole, lysosome, vacuolar membrane, lysosomal membrane), suggesting functions in protein trafficking and recycling. This is supported by an unexpected Gene Ontology for Biological Process (GO:BP) enrichment for glucuronate metabolism, a pathway involved in drug metabolism through linkage of glycosidic bonds [23]. REACTOME pathway analysis [24] supports this enrichment (figure 2*b*). Together, these categories suggest an enhancement of processes involved in clearance, however it is not clear what the underlying mechanism for this enrichment might be.

Considering the downregulated genes (figure 2*b*), GO:CC reveals an enrichment of factors involved in polarized growth and morphogenesis (growth cone, site of polarized growth, distal axon). GO:BP supports this (tube morphogenesis, anatomical structure morphogenesis, animal organ morphogenesis) and more specifically points to tube and vessel development (circulatory system development, blood vessel development, vasculature development). Additionally, GO:BP reveals an enrichment for factors involved in sterol metabolism. We note that some of the genes involved in this category (*Vldlr, Dhcr, Hmgcr, Cyp57*) also appear in the categories associated with vessel development (see electronic supplementary material, file S2). The enrichment for steroid metabolism is supported by REACTOME pathway analysis, which also reveals enrichment for factors involved in ECM organization and integrin–cell surface interactions, pointing to cell–ECM communication.

The *Padi4-*dependent loss of expression of factors that regulate blood vessel development suggested that PADI4 may regulate differentiation into mesodermal and endothelial lineages and prompted us to examine whether *Padi4* depletion affects the complement of cell types present during EB differentiation.

### (d) Single-cell transcriptomic analysis of *Padi4-KO* embryoid bodies reveals a loss of mesodermal differentiation

**Figure 3.**
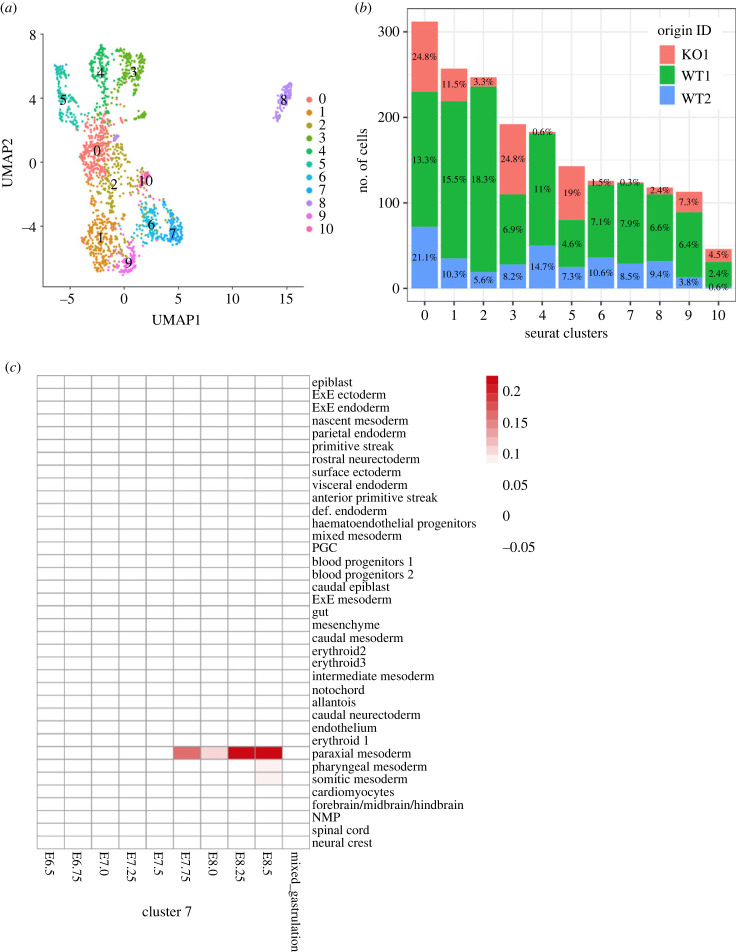

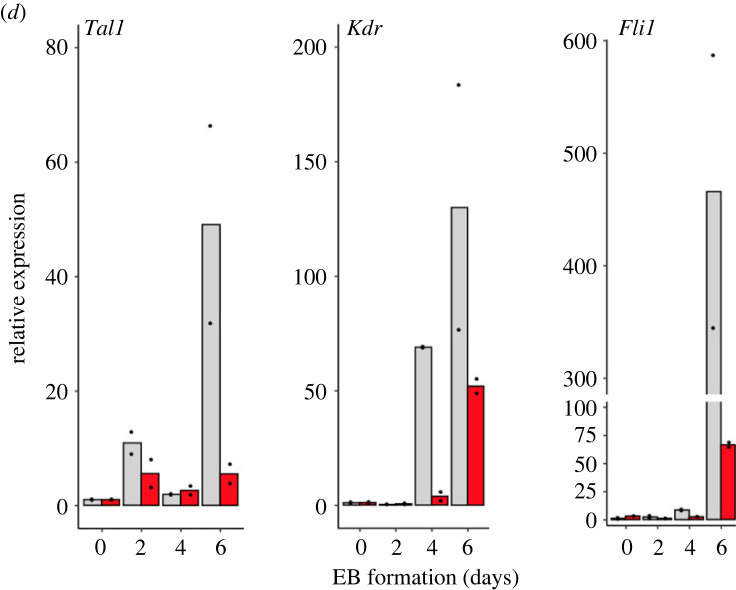
Assessment of the cell types depleted after *Padi4* loss. (*a*) Cell clusters identified in the single-cell RNA sequencing (scRNA-seq) analysis of WT and *Padi4-KO* day 6 EB cells. (*b*) Representation of WT and *Padi4-KO* cells within the scRNA-seq clusters. The percentage of cells of each genotype within each cluster is shown within the bars. (*c*) Cell types represented within Cluster 7, as compared to all cell types and stages of *in vivo* mouse gastrulation [5]. (*d*) RT-qPCR analysis of *Tal1*, *Kdr* and *Fli1* expression in WT (grey) and *Padi4-KO* (red) clonal mouse ES cell lines at the naive state (day 0) and after 2, 4 and 6 days of EB differentiation. Expression is relative to the geometric mean of *Ubqc*, *Gapdh* and *Atp5b*. Statistical analysis was not performed as inappropriate for two biological replicates.

To test whether PADI4 promotes lineage-specific differentiation, we conducted single-cell RNA sequencing analyses of WT and *Padi4-KO* EBs at day 6 of differentiation, using pools of WT or *Padi4-KO* EBs. We identified 10 discernable cell population clusters based on gene expression (figure 3*a*). Assessing the enrichment of each cluster in the WT and *Padi4-KO* cell populations revealed that some of the clusters were under-represented in the *Padi4-KO* EBs, with Cluster 7 being devoid of *Padi4-KO* cells and Clusters 4, 6 and 8 being highly depleted (figure 3*b*). Considering the genes represented in cells within Cluster 7, we generated scores for the different cell types represented in mouse gastrulation. This revealed a strong representation for cells of the mesodermal lineage, specifically paraxial mesoderm, with some representation in somitic and pharyngeal mesoderm (figure 3*c*). Further analysis of the clusters depleted in *Padi4-KO* EBs showed that Clusters 4 and 6 are strongly identified as cardiomyocytes, while Cluster 8 is identified as endothelium (electronic supplementary material, figures S7–S9).

To corroborate the conclusions of the above analyses, we used qRT-PCR to measure the expression of key regulators of mesodermal, cardiomyocyte and endothelial specification in WT and *Padi4-KO* samples during the time course of EB differentiation, in independent experiments (figure 3*d*). We find that *Padi4* loss leads to impairment in *Tal1* upregulation at day 6, with strong downregulation by day 6. Similarly, *Padi4-KO* EBs are impaired in the differentiation-dependent upregulation of Vascular Endothelial Growth Factor Receptor 2 (*Kdr)* and the ETS transcription factor Friend of Leukemia Virus Integration 1 *(Fli1)*, which mark endocardial and endothelial cells, from the earliest time points (day 4).

Collectively, the above results indicate that PADI4 supports mesoderm specification during early embryonic development and may have a role in cardiomyocyte and endothelial differentiation.

## 3. Discussion

In this study we exploited the high resolution afforded by the single-cell transcriptomic atlas of mouse gastrulation [5] to gain insight into the expression of the *Padi* family genes during the critical window of early embryonic lineage specification. The main focus of this study was to understand whether PADI4, which was previously shown to be associated with the pluripotent state in pre-implantation development and during cell reprogramming [10], has a role in regulating the differentiation potential of pluripotent cells. To achieve this, we used CRISPR-mediated genetic perturbation to delete *Padi4* in mouse ES cells and studied the effect of this perturbation on the transcriptional changes associated with multi-lineage differentiation. Finally, we mapped *Padi4-*dependent transcriptional changes onto the mouse gastrulation atlas to understand the lineages affected by *Padi4* loss. Collectively our results indicate a role for PADI4 in mesoderm specification and in cardiomyocyte and endothelial differentiation in particular.

We observed minimal gene expression changes in naive pluripotent cells and widespread changes in EBs. The fact that the *Padi4* gene itself is strongly downsregulated upon EB differentiation indicates that the presence of PADI4 at the naive pluripotency stage has long-lasting consequences during differentiation, even in the absence of continuous gene expression at that stage. Two potential reasons may underlie this observation. It is possible that many of the gene expression changes observed at the EB stage are indirect consequences of *Padi4* loss and downstream of direct PADI4 targets. In this respect, the downregulation of *Tal1,* a key regulator of mesoderm specification [5], may be operative in the observed gene expression changes. Alternatively, PADI4 may contribute to the epigenetic changes that favour mesodermal specification, through post-translational modification (citrullination) of histones, transcription factors or epigenetic regulators [6,10,25,26]. It is worth noting that PADI4 acts as a co-regulator of Tal1 in leukaemia, where it counteracts transcriptionally repressive histone arginine methylation via histone citrullination. It is conceivable that a similar mechanism operates in early development.

At later stages of gastrulation, we observe a loss of cell identities that best correspond to endothelial cells and cardiomyocytes in the mouse gastrulation atlas. These cell identities are the earliest to differentiate from mesoderm and this finding therefore further supports the link between PADI4 and the mesodermal lineage. Gene Ontology analyses have revealed that genes that show *Padi4*-dependent downregulation are, among other categories, associated with the ECM and cell–ECM interactions. This suggests that *Padi4*-*KO* cells are aberrant in ECM deposition. Alternatively, PADI4 may modulate cell signalling and ECM properties through citrullination of matrisomal proteins, as suggested previously [27], and this may result in the observed changes in ECM-associated genes.

The transcriptomic data presented here also give an unbiased overview of the types of genes that are regulated by PADI4 in non-pathological conditions. To our knowledge, this is the first dataset of this type. We were surprised to find a strong enrichment, within the upregulated factors, in processes associated with vacuoles, lysosomes and associated membranes. These gene categories, in addition to genes associated with the ECM, may collectively point to functions that underlie cell–ECM communication. Further work is needed to understand the mechanistic and functional significance of these findings.

Although this study has focused on early events in embryonic cell differentiation, the general principles identified here may translate across different aspects of development and tissue homeostasis. The data presented here may therefore serve as a primer for future work that will determine the physiological functions of PADI4.

## 4. Methods

### (a) Cell culture

J1 mouse ES cells were cultured on tissue culture dishes pre-coated with 0.1% gelatine in 2i + LIF medium (GMEM (BHK21) medium supplemented with 10% KnockOut Serum replacement, 1% fetal bovine serum, 0.1 mM 2-mercaptoethanol, 0.1 mM non-essential amino acids, 1 mM sodium pyruvate, 2 mM l-glutamine, 50 ng ml^−1^ LIF, MEK inhibitor PD0325901 (1 µM) and GSK3 inhibitor CHIR99021 (3 µM). For the EB formation, 1 million J1 mESCs cells were seeded in EB media (DMEM, 15% fetal bovine serum, 0.1 mM non-essential amino acids, 1 mM sodium pyruvate, 0.1 mM 2-mercaptoethanol, 2 mM l-glutamine) in 10 cm low attachment plates (Greiner Bio-One, 633102). EB media was changed every 48 h by transferring cell suspensions to 15 ml Falcon tubes and leaving at room temperature for 10 min to settle. Cells were harvested at different time points for downstream analyses.

### (b) PADI4 knockout using CRISPR-Cas9

J1 mESCs were transiently co-transfected using Lipofectamine 2000, following the manufacturer's instructions, with a pCAG-Cas9-GFP plasmid expressing Cas9 and GFP and gBlock (Integrated DNA Technology) containing U6 promoter and *Padi4* targeting sequences (CACCCGCGGACATCCAGCGGGG) in exon 1 in 1 : 3 ratio, respectively. After 48 h of transfection, GFP-positive cells were single-cell sorted in 96 well plate using a fluorescence-activated cell sorter (FACS). After 7 days, at which point transient expression of Cas9 and GFP no longer persisted, colonies were accessed and propagated individually and screened by western blotting and genomic DNA PCR using PCR primers: PADI4_Scr_F – TCTTCTGCTGTTGCAGGCTT and PADI4_Scr_R – TAATTGGCACGATAGGCCCC pCAG-Cas9-GFP [28] was a gift from Kiran Musunuru (Addgene, http://n2t.net/addgene:44719).

### (c) RT-qPCR

RNA was isolated using the RNeasy Mini Kit (Qiagen, 74104) according to the manufacturer’s instructions. One microgram of RNA was used for cDNA synthesis using the Qiagen Reverse-Transcription kit (Qiagen, 205311). After 1 : 100 dilution of the resulting cDNA, qRT-PCR was performed in triplicate using the SYBR green JumpStart Readymix (Sigma, S5193). The comparative ∆∆Ct method was used to quantify the relative levels of genes of interest relative to the geometric mean of *Gapdh*, *UbqC* and *Atp5b*. The primers were used at 200 nM concentration, and their sequences are provided below. *Tal1_ Forward*: ACAACAACCGGGTGAAGAGG; *Tal1_Reverse*: ACTTTGGTGTGAGGACCATC; *Kdr_Forward*: TTCCATGTGATCAGGGGTCC; *Kdr_Reverse*: ACTGGTGTGAGTGATTCGCC; *Fli1_Forward*: CTCTGGCCTCAACAAAAGTCC; *Fli1­Reverse*: TTTGAACTCCCCGTTGGTCC; *Padi4_Forward*: TCCTCCAGTCAAGAAGAGTACCAT; *Padi4_Reverse*: GTCCATAGTATGAAACTCGAACCTT; *UbqC_Forward*: GAGTTCCGTCTGCTGTGTGA; *UbqC_Reverse*: TCACAAAGATCTGCATCGTCA; *Atp5b_Forward*: GGCCAAGATGTCCTGCTGTT; *Atp5b_Reverse*: GCTGGTAGCCTACAGCAGAAGG; *Gapdh_Forward*: CTCCCACTCTTCCACCTTCG; *Gapdh_Reverse*: GCCTCTCTTGCTCAGTGTCC.

### (d) Immunoblotting

Cell lysates were prepared after scraping the cells in RIPA buffer (25 mM Tris-cl (pH 7.4), 150 mM NaCl, 1% NP-40, 0.1% SDS, 0.5% sodium deoxycholate, 1 mM EDTA) supplemented with protease inhibitors (Roche, UK). Lysates were sonicated for five times, 10 s each, at 10 amplitudes and were cleared by centrifugation and quantified using the BCA reagent (Thermo). Normalized amounts of total protein were resolved with SDS-PAGE and analysed by immunoblotting with anti-PADI4 (Abcam, ab241810, 1 : 1000) and anti-GAPDH (Abcam, Ab8245, 1 : 5000) antibodies.

### (e) RNA sequencing and analysis

RNA was isolated using AllPrep DNA/RNA Mini Kit (Qiagen, 80204) following the manufacturer's protocol. RNA sequencing including library preparation was carried out by Novogene Europe using 150-bp paired-end reads. Raw FASTQ files were subject to initial quality control analyses using the FASTQC software (https://www.bioinformatics.babraham.ac.uk/projects/fastqc/). FASTQ files were then trimmed using Trim-galore (https://www.bioinformatics.babraham.ac.uk/projects/trim_galore/) and aligned to the mouse genome (Grcm38) using hisat2 [29]. The Seqmonk program (https://www.bioinformatics.babraham.ac.uk/projects/seqmonk/) was used to generate raw counts and differential gene expression performed using DESEq2 [30]. PCA, volcano and MA plots were generated in R using ggplot2. Genes were filtered on padj < 0.05 and FC > 1.5 and heat-map analyses performed using top 60 differentially expressed genes (30 upregulated and 30 downregulated) which were clustered using the complete agglomeration method. For the generation of the heat map, the gene expression data were *z*-score normalized, [z−score=(x− mean(x))/(s.d.(x))], where x represents raw counts.

### (f) Gene Ontology analyses

For GO analysis, differentially expressed genes were sorted based on log2FC and a ranked analysis was performed using the gProfiler software [31]. All genes expressed in the cells ware used as the custom background for the analysis. Ranked GO analyses were performed separately for upregulated and downregulated genes.

### (g) Single-cell RNA sequencing

CellRanger v. 7.0.0 was used to process 10X single-cell RNA data using the 10X mm10-2020-A transcription as reference. Downstream analysis was performed in R using Seurat v. 4.1.1. The filtered count tables from CellRanger were imported and merged. Data were quantitated using the Cell-wise Centred Log Ratio method and then scaled and centred into z-scores. PCA was performed on the 2000 most variable features, which showed that PC1 divided by total read count. Further steps in the analysis proceeded using PCs 2 to 10. Dimension reduction projection was performed using UMAP using default parameters. Clustering was performed using Louvain clustering.

Enrichment of *Padi4-KO* cells in clusters was calculated as the log2 ratio of the proportion of KO and WT cells falling in to each cluster. Enrichment was assessed statistically using Fisher's exact test. Markers for single-cell clusters were calculated using a Wilcoxon rank sum test with a false discovery cut-off of 0.05 and average log2 fold change of ≥0.6.

Gastrulation single-cell data were taken from the MouseGastrulationData Bioconductor package [5]. Cluster markers were used to construct a combined module score method using the Seurat AddModuleScore function, and this was applied to the pre-normalized gastrulation data. The scores for all cells were projected onto the pre-calculated UMAP coordinates, and were summarized per cell type as defined in the original data.

## Data Availability

The data are provided in electronic supplementary material, file S1 and electronic supplementary material, file S2 [32].

## References

[1] Tam PPL, Behringer RR. 1997 Mouse gastrulation: the formation of a mammalian body plan. Mech. Dev. **68**, 3-25. (10.1016/S0925-4773(97)00123-8) 9431800

[2] Peng G et al. 2016 Spatial transcriptome for the molecular annotation of lineage fates and cell identity in mid-gastrula mouse embryo. Dev. Cell **36**, 681-697. (10.1016/j.devcel.2016.02.020) 27003939

[3] Mohammed H et al. 2017 Single-cell landscape of transcriptional heterogeneity and cell fate decisions during mouse early gastrulation. Cell Rep. **20**, 1215-1228. (10.1016/j.celrep.2017.07.009) 28768204 PMC5554778

[4] Wen J et al. 2017 Single-cell analysis reveals lineage segregation in early post-implantation mouse embryos. J. Biol. Chem. **292**, 9840-9854. (10.1074/jbc.M117.780585) 28298438 PMC5465504

[5] Pijuan-Sala B et al. 2019 A single-cell molecular map of mouse gastrulation and early organogenesis. Nature **566**, 490-495. (10.1038/s41586-019-0933-9) 30787436 PMC6522369

[6] Christophorou MA. 2022 The virtues and vices of protein citrullination. R. Soc. Open Sci. **9**, 220125. (10.1098/rsos.220125) 35706669 PMC9174705

[7] Sun B et al. 2017 Citrullination of NF-B p65 promotes its nuclear localization and TLR-induced expression of IL-1 and TNF. Sci. Immunol. **2**, eaal3062. (10.1126/sciimmunol.aal3062) 28783661 PMC5718838

[8] Ghari F et al. 2016 Citrullination-acetylation interplay guides E2F-1 activity during the inflammatory response. Sci. Adv. **2**, e1501257. (10.1126/sciadv.1501257) 26989780 PMC4788482

[9] Wang Y et al. 2009 Histone hypercitrullination mediates chromatin decondensation and neutrophil extracellular trap formation. J. Cell Biol. **184**, 205-213. (10.1083/jcb.200806072) 19153223 PMC2654299

[10] Christophorou MA et al. 2014 Citrullination regulates pluripotency and histone H1 binding to chromatin. Nature **507**, 104-108. (10.1038/nature12942) 24463520 PMC4843970

[11] Chang X, Han J, Pang L, Zhao Y, Yang Y, Shen Z. 2009 Increased PADI4 expression in blood and tissues of patients with malignant tumors. BMC Cancer **9**, 1-11. (10.1186/1471-2407-9-40) 19183436 PMC2637889

[12] Kolodziej S et al. 2014 PADI4 acts as a coactivator of Tal1 by counteracting repressive histone arginine methylation. Nat. Commun. **5**, 3995. (10.1038/ncomms4995) 24874575 PMC4050257

[13] Nakashima K et al. 2013 PAD4 regulates proliferation of multipotent haematopoietic cells by controlling c-myc expression. Nat. Commun. **4**, 1836. (10.1038/ncomms2862) 23673621 PMC3674250

[14] Uhlén M et al. 2015 Tissue-based map of the human proteome. Science **347**, 1260419. (10.1126/science.1260419) 25613900

[15] Young C, Russell JR, Van De Lagemaat LN, Lawson H, Mapperley C, Kranc KR, Christophorou MA. 2022 Intrinsic function of the peptidylarginine deiminase PADI4 is dispensable for normal haematopoiesis. Biol. Open **11**, bio059143. (10.1242/bio.059143) 35603697 PMC9212077

[16] Argelaguet R et al. 2019 Multi-omics profiling of mouse gastrulation at single cell resolution. Nature **576**, 487-491. (10.1038/s41586-019-1825-8) 31827285 PMC6924995

[17] Doetschman TC, Eistetter H, Katz M. 1985 The *in vitro* development of blastocyst-derived embryonic stem cell lines: formation of visceral yolk sac, blood islands and myocardium. J. Embryol. Exp. Morphol. **87**, 27-45. (10.1242/dev.87.1.27) 3897439

[18] Méchin MC, Takahara H, Simon M. 2020 Deimination and peptidylarginine deiminases in skin physiology and diseases. Int. J. Mol. Sci. **21**, 566. (10.3390/ijms21020566) 31952341 PMC7014782

[19] Zhang X, Liu X, Zhang M, Li T, Muth A, Thompson PR, Coonrod SA, Zhang X. 2016 Peptidylarginine deiminase 1-catalyzed histone citrullination is essential for early embryo development. Sci. Rep. **6**, 38727. (10.1038/srep38727) 27929094 PMC5144008

[20] Ballasy NN, Bering EA, Kokorudz C, Radford BN, Zhao X, Dean W, Hemberger M. 2022 Padi2/3 deficiency alters the epigenomic landscape and causes premature differentiation of mouse trophoblast stem cells. Cells **11**, 2466. (10.3390/cells11162466) 36010543 PMC9406452

[21] Esposito G, Vitale AM, Leijten FPJ, Strik AM, Koonen-Reemst AMCB, Yurttas P, Robben TJAA, Coonrod S, Gossen JA. 2007 Peptidylarginine deiminase (PAD) 6 is essential for oocyte cytoskeletal sheet formation and female fertility. Mol. Cell. Endocrinol. **273**, 25-31. (10.1016/j.mce.2007.05.005) 17587491

[22] Gao Y et al. 2017 Protein expression landscape of mouse embryos during pre-implantation development. Cell Rep. **21**, 3957-3969. (10.1016/j.celrep.2017.11.111) 29281840

[23] Miners JO, Mackenzie PI. 1991 Drug glucuronidation in humans. Pharmacol. Ther. **51**, 347-369. (10.1016/0163-7258(91)90065-T) 1792239

[24] Sidiropoulos K et al. 2017 Reactome enhanced pathway visualization. Bioinformatics **33**, 3461-3467. (10.1093/bioinformatics/btx441) 29077811 PMC5860170

[25] Zhang X, Gamble MJ, Stadler S, Cherrington BD, Causey CP, Thompson PR, Roberson MS, Kraus WL, Coonrod SA. 2011 Genome-wide analysis reveals PADI4 cooperates with Elk-1 to activate C-Fos expression in breast cancer cells. PLoS Genet. **7**, e1002112. (10.1371/journal.pgen.1002112) 21655091 PMC3107201

[26] Sharma P, Azebi S, England P, Christensen T, Møller-Larsen A, Petersen T, Batsché E, Muchardt C. 2012 Citrullination of histone H3 interferes with HP1-mediated transcriptional repression. PLoS Genet. **8**, e1002934. (10.1371/journal.pgen.1002934) 23028349 PMC3441713

[27] Sipilä KH, Ranga V, Rappu P, Torittu A, Pirilä L, Käpylä J, Johnson MS, Larjava H, Heino J. 2016 Extracellular citrullination inhibits the function of matrix associated TGF-β. Matrix. Biol. **55**, 77-89. (10.1016/j.matbio.2016.02.008) 26923761

[28] Ding Q, Regan S, Xia Y, Oostrom L, Cowan C, Musunuru K. 2013 Enhanced efficiency of human pluripotent stem cell genome editing through replacing TALENs with CRISPRs. Cell Stem Cell **12**, 393-394. (10.1016/j.stem.2013.03.006) 23561441 PMC3925309

[29] Kim D, Paggi JM, Park C, Bennett C, Salzberg SL. 2019 Graph-based genome alignment and genotyping with HISAT2 and HISAT-genotype. Nat. Biotechnol. **37**, 907-915. (10.1038/s41587-019-0201-4) 31375807 PMC7605509

[30] Love MI, Huber W, Anders S. 2014 Moderated estimation of fold change and dispersion for RNA-seq data with DESeq2. Genome Biol. **15**, 1-21. (10.1186/s13059-014-0550-8) PMC4302049 25516281

[31] Raudvere U, Kolberg L, Kuzmin I, Arak T, Adler P, Peterson H, Vilo J. 2019 G:Profiler: a web server for functional enrichment analysis and conversions of gene lists (2019 update). Nucleic Acids Res. **47**, W191-W198. (10.1093/nar/gkz369) 31066453 PMC6602461

[32] Singh AK, Khan S, Moore D, Andrews S, Christophorou MA. 2023 Transcriptomic analysis of PADI4 target genes during multi-lineage differentiation of embryonic stem cells. Figshare. (10.6084/m9.figshare.c.6806493) PMC10542446 37778387

